# Correction: Sturm et al. Exposure of Bladder Cancer Cells to Blue Light (λ = 453 nm) in the Presence of Riboflavin Synergistically Enhances the Cytotoxic Efficiency of Gemcitabine. *Int. J. Mol. Sci*. 2024, *25*, 4868

**DOI:** 10.3390/ijms26104790

**Published:** 2025-05-16

**Authors:** Sofia Sturm, Günter Niegisch, Joachim Windolf, Christoph V. Suschek

**Affiliations:** 1 Department of Orthopedics and Trauma Surgery, Medical Faculty, University Hospital Düsseldorf, Heinrich-Heine-University Düsseldorf, Moorenstr. 5, 40225 Düsseldorf, Germany; sofia.sturm@hhu.de (S.S.); windolf@hhu.de (J.W.); 2 Department of Urology, Medical Faculty, University Hospital Düsseldorf, Heinrich-Heine-University Düsseldorf, 40225 Düsseldorf, Germany; guenter.niegisch@med.uni-duesseldorf.de

## Figure Legend

In the original publication [1], there was a mistake in the legend for Figures 2–5. The concentration unit for Gemcitabine should be ng/mL, and the assay we exclusively utilized is the MTT assay for detecting living cells, but in the original article, the concentration unit shows µM and the assay shows CTB.

The correct legend appears below.

**Figure 2.** Impact of blue light exposure on viability of urothelial carcinoma cells. Urothelial carcinoma cell line cultures (BFTC-905; RT-112; SW-1710) were irradiated with blue light (l = 453 nm) at light doses indicated (50, 80,110, 140, 200, 300 J/cm^2^). Twenty-four hours after light exposure, the number of living cells was detected by MTT assay. Bars represent the mean ± S.D, normalised to the value of the non-treated controls of four individual experiments (n = 4). Statistical evaluation (paired *t*-test) was carried out with the values of the original measurements, i.e., before the normalisation process to the respective control value shown here (contr.). *, *p* < 0.05 compared with the respective non-irradiated cell cultures.
**Figure 3.** Impact of blue light-activated riboflavin on viability of exposed urothelial carcinoma cell cultures. Urothelial carcinoma cell line cultures (**A**) BFTC-905; (**B**) RT-112; (**C**) SW-1710 were exposed to blue light (grey bars, 110 J/cm^2^) in the absence (0 µM) or presence of 5, 10, 20 or 25 µM riboflavin. White bars represent the values of the respective non-irradiated control cultures. Twenty-four hours after light exposure, the number of living cells was detected by MTT assay. Bars represent the mean ± S.D, normalised to the value of the non-treated controls of eight individual experiments (n = 8). The statistical evaluation (two-way ANOVA) was carried out with the values of the original measurements, i.e., before the normalisation process to the respective control value shown here (contr.). *, *p* < 0.05 compared with the value of the respective non-irradiated control cultures.
**Figure 4.** Impact of blue light-activated riboflavin on the cytotoxic capacity of gemcitabine. Urothelial carcinoma cell line cultures ((**A**) BFTC-905; (**B**) RT-112; (**C**) SW-1710) were maintained in the absence or presence of 10 or 25 ng/mL gemcitabine (contr., white bars). Additionally, these cultures were incubated with 10 µM riboflavin (10 µM RF, black bars). Finally, control cultures (contr., white bars) were exposed to blue light with a dose of 110 J/cm^2^ (+453 nm, grey bars,) and the riboflavin-containing cultures (10 µM RF, black bars) also were exposed to blue light with a dose of 110 J/cm^2^ (10 µM RF + 453 nm, lined bars). Twenty-four hours after light exposure, the number of living cells was detected by MTT assay. Bars represent the mean ± S.D, normalised to the value of the non-treated controls, of seven individual experiments (n = 7). The statistical evaluation (two-way ANOVA) was carried out with the values of the original measurements, i.e., before the normalisation process to the respective control value shown here (contr.). *, *p* < 0.05 compared with the respective cultures irradiated in the absence of riboflavin (grey bars); #, *p* < 0.05 compared with the respective control cultures (contr., white bars); §, *p* < 0.05 compared with the respective non-irradiated riboflavin-containing cultures (10 µM RF, black bars); $, *p* < 0.05 compared with the respective control cultures maintained in the absence of gemcitabine (white bars, 0 ng/mL gemcitabine).
**Figure 5.** Impact of blue light-activated riboflavin on lipid peroxidation and mitochondrial respiratory chain activity of urothelial carcinoma cell cultures. (**A**), Urothelial carcinoma cells (BFTC-905) were maintained in the absence or presence of 10, 25 or 50 ng/mL gemcitabine (contr., white bars). Additionally, these cultures were incubated with 10 µM riboflavin (10 µM RF, black bars). Finally, control cultures (contr., white bars) were exposed to blue light with a dose of 110 J/cm^2^ (+453 nm, grey bars,) and the riboflavin-containing cultures (10 µM RF, black bars) also were exposed to blue light with a dose of 110 J/cm^2^ (10 µM RF + 453 nm, lined bars). Twenty-four hours after light exposure, the concentration of malondialdehyde as a lipid peroxidation marker was quantified using a specific assay. Bars represent the mean ± S.D, normalised to the value of the non-treated controls, of three individual experiments (n = 3). The statistical evaluation (paired *t*-test) was carried out with the values of the original measurements, i.e., before the normalisation process to the respective control value shown here (contr.). *, *p* < 0.05 compared with all other cultures; #, *p* < 0.05 compared with the respective control cultures (contr., white bars). Additionally, key parameters of the respiratory chain, basal respiration (**B**), ATP production rate (**C**), maximal respiration (**D**) and relative spare respiration capacity (**E**) of non-irradiated (white bars) as well as blue light exposed (BL, 110 J/cm^2^) BFTC-905, RT-112 or SW-1710 tumour cell cultures were detected. The statistical evaluation was carried out with the values of the original measurements. *, *p* < 0.05 compared with the respective values obtained with the non-irradiated cultures.

## Text Correction

There were some errors in the original publication [1]. The concentration unit for Gemcitabine is partly incorrect and assay methods were incorrectly written. The concentration unit for Gemcitabine should be ng/mL. And in all other cases where no irradiation was performed, we used the CTB assay, as it can be performed more quickly and securely. But in the original article, the concentration unit shows µM and the assay shows MTT. A correction has been made to 2. Results, 2.1 Evaluation of the Cytotoxic Capacity of Gemcitabine, paragraphs 1 and 2, and 3. Discussion, paragraphs 3–6.

The authors state that the scientific conclusions are unaffected. This correction was approved by the Academic Editor. The original publication has also been updated.

## References

[1] Sturm S., Niegisch G., Windolf J., Suschek C.V. (2024). Exposure of Bladder Cancer Cells to Blue Light (λ = 453 nm) in the Presence of Riboflavin Synergistically Enhances the Cytotoxic Efficiency of Gemcitabine. Int. J. Mol. Sci..

